# Fungal bioproducts: An annotated selection of World Wide Web sites relevant to the topics in *environmental microbiology*

**DOI:** 10.1111/1751-7915.12331

**Published:** 2015-10-20

**Authors:** Lawrence P Wackett

**Affiliations:** 1 Department of Biochemistry, Molecular Biology and Biophysics, BioTechnology Institute, University of MinnesotaSt Paul, MN, 55108, USA

## Industrial uses of fungi

### https://www.emlab.com/s/sampling/env-report-09-2006.html

This article describes some of the most commercially important fungi, and focuses on food products.

## Strategies for mining fungal natural products

### http://www.ncbi.nlm.nih.gov/pubmed/24146366

This general review article discusses all different aspects of fungal natural products, from pharmaceuticals to food toxins.

## Fungi in designer products

### http://www.dezeen.com/2015/01/21/movie-officina-corpuscoli-growing-products-materials-fungus-biotechnological-revolution/

This website shows novel fungal structures that can have useful properties as novel everyday materials, largely for home use.

## Synthetic biology of fungal natural products

### http://journal.frontiersin.org/article/10.3389/fmicb.2015.00775/abstract

Synthetic biology has been applied largely to manipulations with bacteria and yeast and is increasingly being applied to filamentous fungi.

## Fungi and yeast: ATCC

### http://www.atcc.org/en/Products/Cells_and_Microorganisms/Fungi_and_Yeast.aspx

This website contains links to information and access to a large collection of cultures of fungi and yeast.

## Fungal databases

### http://nt.ars-grin.gov/fungaldatabases/

This page contains links and searches for information on different classes of fungi.

## Mycobank database

### http://www.mycobank.org

This database focuses on the identification and nomenclature of different fungi. It contains sequence alignments that can be helpful to users.

## Ensembl fungi

### http://fungi.ensembl.org/info/website/ftp/index.html

This website contains information on fungal genomes and blast results from genome analysis.

## Fungal genomics

### https://www.broadinstitute.org/scientific-community/science/projects/fungal-genome-initiative/fungal-genomics

This site contains information on over 100 fungal genomes, highlighted due to medical, agricultural, commercial, or fundamental science reasons.

## Database of fungal secondary metabolites

### http://pubs.acs.org/doi/abs/10.1021/np4004307

This paper deals with the cataloguing chemical identification data for fungal metabolites in an effort to avoid rediscovering already known natural products in searching for new products.

## Fungal secretome knowledge base

### http://proteomics.ysu.edu/secretomes/fungi.php

This website provides information on fungal secreted proteins, also called the secretome. There are a significant number of fungal secreted enzymes that are useful industrially.

## Antibiotics database

### https://www.tebu-bio.com/search/Molecules/Antibiotics_;_Metabolites

This commercial website contains a large list of natural products, many from fungi, and so it provides a useful compendium.

## Drugs from fungi

### http://bugs.bio.usyd.edu.au/learning/resources/Mycology/UsesOf_Fungi/industrialProduction/fungalDrugs.shtml

This page lists and gives some detail on the most prominent pharmaceutical products derived from fungi.

## Fungal jewels: Secondary metabolites

### http://journal.frontiersin.org/researchtopic/2740/fungal-jewels-secondary-metabolites

This collection of articles deals with the diversity of chemical structures made by fungi with a focus on compounds that have valuable bioactive properties.

## Putting fungi to work

### http://journals.plos.org/plospathogens/article?id=10.1371/journal.ppat.1003950

These interesting review articles detail the many natural products produced by fungi, some of which are very toxic, whereas others are extremely useful.

## Fungal biotechnology: JBEI

### http://www.jbei.org/research/divisions/deconstruction/fungal-biotechnology/

This webpage briefly describes projects on fungal biotechnology related to the production of biofuels.

## Fungi in biotechnology for fuels

### http://www.biotechnologyforbiofuels.com/content/8/1/38

A large number of fungi isolated from the surface of a common biofuels feedstock plant were tested for excreted enzymes that degrade the plant biomass.

